# Supplementary material for: Immunoscore Combining CD8, FoxP3, and CD68-Positive Cells Density and Distribution Predicts the Prognosis of Head and Neck Cancer Patients
Source: Cells. 2022 Jun 28;11(13):2050. doi: 10.3390/cells11132050 (PMC9266282; doi:10.3390/cells11132050)
Supplement: Supplementary file 1 [file cells-11-02050-s001.zip › cells-1757592-supplementary.pdf]

Table S1. Description of immunostaining experimental condition.

**Immunostaining**	**CD8**	**FoxP3**	**CD68**
	Antigen retrieval		
Buffers	EDTA 10%	Citrate 10%	EDTA 10%
Conditions	MP 6min	MW 21min	MP 6min
Blocage	Caseine 0,5% 15min	CSAII Kit, Dako	Caseine 0,5% 15min
	Primary Ab		
Speces	Mouse	Mouse	Mouse
Manufacturer	Dako	Invitrogen	Dako
Dilution	1/200	1/200	1/200
Timing	1h RT	1h RT	1h RT
	Secondary Ab		
References	BrightVision+ Poly- HRP-Anti Mouse/Rabbit IgG, VWR	CSAII Kit, Dako	BrightVision+ Poly- HRP-Anti Mouse/Rabbit IgG, VWR

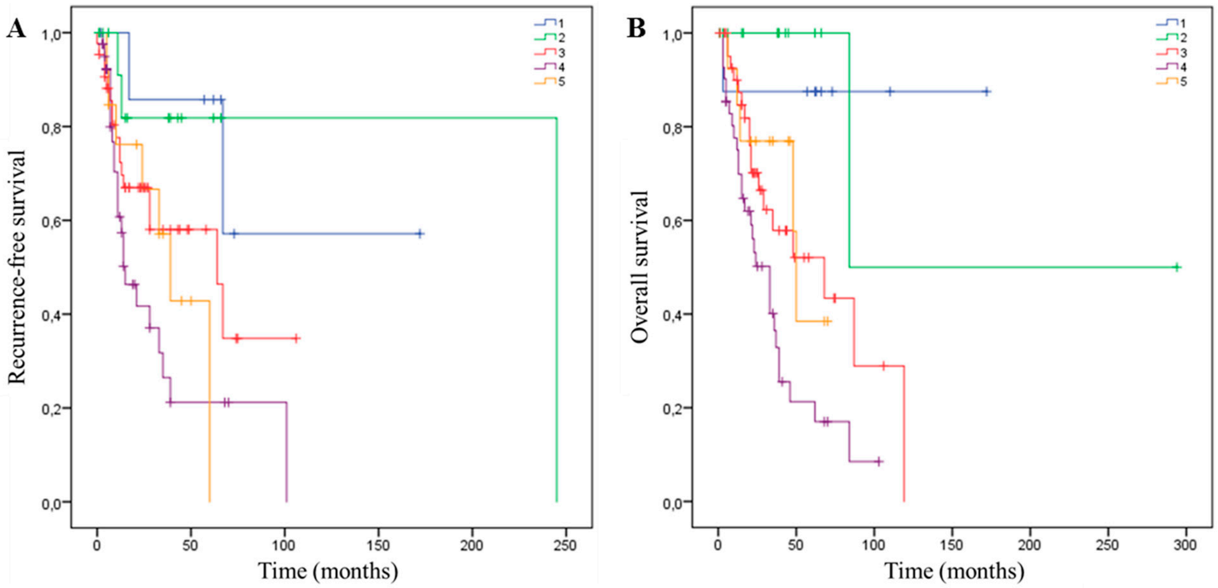

Figure S1. Immunoscore included CD8 ST/IT, FoxP3 ST/IT and CD68 IT. (**A**) Kaplan Meier curves comparing recurrence-free survival (RFS) and (**B**) overall survival (OS) of our scoring system (range 1 to 5).

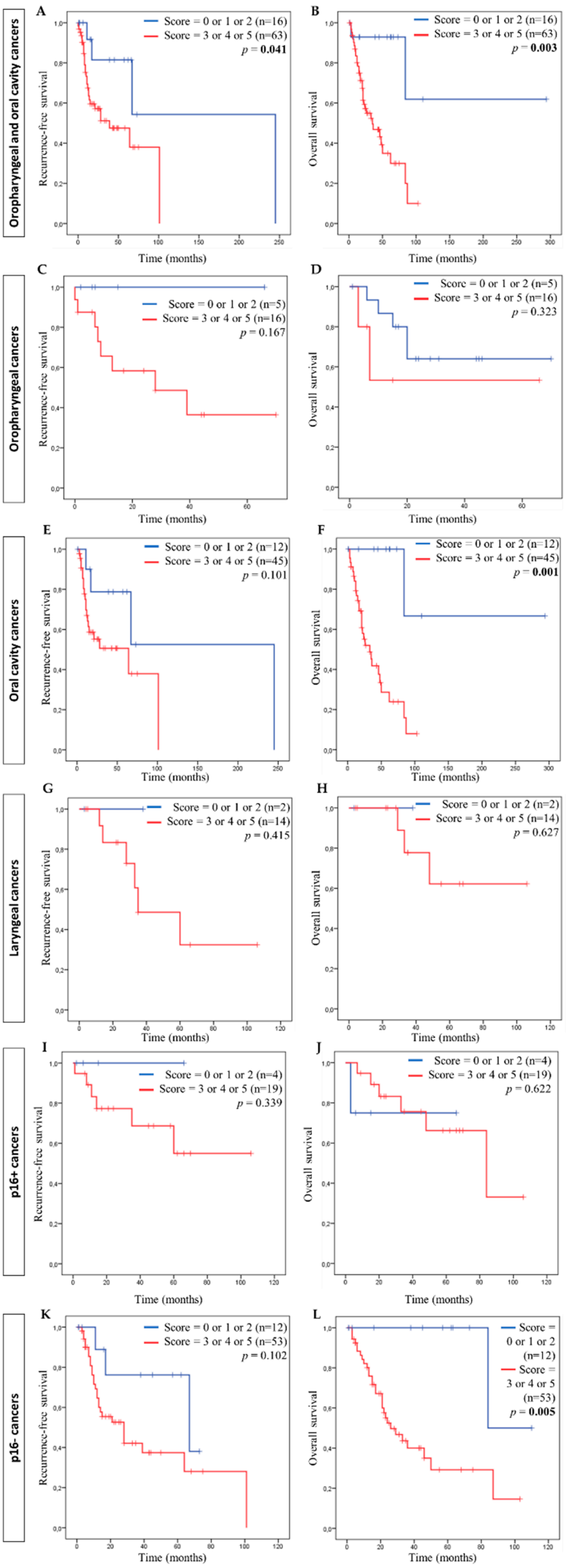

Figure S2. Immunoscore included CD8 ST/IT, FoxP3 ST/IT and CD68 IT. Kaplan Meier curves comparing recurrence-free survival (RFS) and overall survival (OS) of our scoring system (range 1 to 5) in oropharyngeal and oral cavity cancers (**A-B**), in oropharyngeal cancers (**C-D**), in oral cavity cancers (**E-F**), in laryngeal cancers (**G-H**), in p16+ HNSCC (**I-J**), p16- HNSCC (**K-L**).
